# Inequities in effective coverage of family planning services in low- and middle-income countries: linking household and facility surveys

**DOI:** 10.7189/jogh.15.04211

**Published:** 2025-09-12

**Authors:** Shenning Tian, Elizabeth A Hazel, Melinda Munos, Abdoulaye Maïga, Safia S Jiwani, Emily B Wilson, Gouda Roland Mesmer Mady, Agbessi Amouzou

**Affiliations:** Department of International Health, Johns Hopkins Bloomberg School of Public Health, Baltimore, Maryland, USA

## Abstract

**Background:**

Despite decades of family planning (FP) programme successes in low- and middle-income countries (LMICs), women still face an unmet need for contraceptives, as well as inequalities in coverage. Including elements of service readiness in FP intervention coverage measures will better inform population-level programme performance.

**Methods:**

We identified five LMICs that had health facility and household surveys conducted <5 years apart within the past ten years: Bangladesh, Haiti, Malawi, Nepal, and Tanzania; only Nepal had time-trend data available. We developed quality readiness-adjusted FP coverage measures by linking health facility assessments (*i.e.* readiness) and household surveys (*i.e.* intervention coverage) according to ecological linking methods. We defined linking units by facility type, managing authority, and geographic location using women’s reported source of contraceptives. We defined intervention coverage as the percentage of women aged 15–49 years who needed contraceptive services and were using a modern method, and calculated readiness-adjusted intervention coverage with the average FP readiness score in each linking unit. We used a coverage cascade model to understand gaps in health service readiness and access, and performed a health equity analysis for wealth, locality, and age.

**Results:**

Large gaps in FP intervention coverage and readiness were present in all settings. Facility readiness scores ranged from 0.58 to 0.66, with gaps in coverage and readiness-adjusted coverage ranging from 49 percentage points (pp) in Bangladesh to 21 pp in Haiti. Urban, wealthier, and adolescent women had lower readiness-adjusted coverage because they also obtained their contraceptives outside of health facilities. Coverage cascades changed little for Nepal between 2015 and 2021.

**Conclusions:**

By calculating readiness-adjusted FP coverage using a cascade model in five geographically diverse countries, including time trends in one country, we found large gaps in intervention and readiness-adjusted coverage with related inequalities.

Despite substantial improvements in family planning (FP) programmes over the last 50 years, 164 million women of reproductive age worldwide still had an unmet need for contraception in 2021 [1]. Most women facing unmet needs are from low- and middle-income countries, with more than half living in sub-Saharan Africa and South Asia [2].

One important factor that contributes to the unmet need for FP is poor quality of care. A 2015 study reported that 27% of women in low- and middle-income countries cited poor quality of care as the reason for contraception discontinuation [3]. Sufficient quality of FP services enables informed choice of contraceptives, appropriate knowledge of their use, management of their side effects, and continuity of care [4]. According to classic quality-of-care frameworks, quality encompasses the structure of care, the process of care, and outcomes [5]. Structural quality, also called readiness, measures whether the environment of health services is sufficient to provide quality care. Process quality is the standard of care, and outcomes constitute patient-level results, such as knowledge and satisfaction with services. The linkages between these three domains are not linear, likely owing to the complex nature of health service provision [6]. In this study, we focussed on measuring readiness (*i.e.* structural quality) as a necessary but insufficient condition of process quality and improved patient outcomes.

A common indicator of FP service coverage is the proportion of women using contraception among those who need it [7]. However, this indicator does not reflect the quality of services delivered. To address this limitation, some researchers in the field have proposed adding effective coverage measures as quality-of-care elements to better capture the potential health benefits of the intervention [8–10]. Amouzou and colleagues [11] proposed a coverage cascade framework consisting of six steps to measure the potential loss of health benefits at each step. Using this stepwise model, we defined the target population, service contact, intervention coverage, and readiness-adjusted coverage of demand for FP services satisfied at a health facility equipped and ready to provide quality FP services.

We aimed to describe the facility readiness and inequities in readiness-adjusted coverage for FP care in Bangladesh, Haiti, Malawi, Nepal, and Tanzania using a coverage cascade model. In Nepal, where multiple data sets were available, we also assessed trends in readiness and coverage cascade of FP services.

## METHODS

### Data

This study was a secondary analysis of publicly available data from the Service Provision Assessment (SPA) and Demographic and Health Surveys (DHS) Program in Bangladesh, Haiti, Malawi, Nepal, and Tanzania. The SPA is a sample survey or census of health facilities that collects national- and sub-national-level information on the availability and quality of health services from the country [12–17]. The DHS was conducted in a nationally representative sample of households to provide estimates on demographic and health indicators, including access to and use of modern FP methods [18–23]. We selected these five countries because each had the required data from DHS and SPAs that had been completed <5 years apart during a recent ten-year period (**Table 1**). Nepal was the only country with two SPAs and DHS that met these criteria, enabling us to perform a trend analysis.

**Table 1:** Description of the surveys

			Contraceptives currently used (%)								
Country/survey type*	**Survey year**	**Sample size**	**Pill**	**IUD**	**Injections**	**Male condom**	**Sterilisation**	**Implants/norplant**	**Emergency contraception**	**Traditional**	**Other modern†**
**Bangladesh**											
SPA	2017	1353									
DHS	2017-18	18 895	41.00	0.89	17.38	11.61	9.53	3.45	<0.01	16.09	<0.01
**Haiti**											
SPA	2017-18	756									
DHS	2016-17	14 371	5.82	0.25	49.95	25.92	3.28	5.64	0.00	7.59	1.50
**Malawi**											
SPA	2013-14	810									
DHS	2015-16	24 562	3.78	1.75	48.96	5.59	18.27	19.49	<0.01	1.75	0.16
**Nepal**											
SPA	2015	899									
DHS	2016	12 862	8.70	2.68	16.83	8.01	38.84	6.27	0.12	18.43	<0.01
SPA	2021	1478									
DHS	2022	14 845	7.76	2.22	16.04	7.99	30.23	10.47	<0.01	25.12	<0.01
**Tanzania**											
SPA	2014-15	933									
DHS	2015-16	13 265	12.75	2.11	30.58	12.01	7.96	17.14	<0.01	16.34	0.93

DHS – Demographic and Health Survey, IUD – intrauterine device, SPA – Service Provision Assessment

*Data for all surveys except for the Malawi SPA were collected from a sample; data for the Malawi SPA were collected as a census.

†Includes lactational amenorrhea, female condom, standard days method, or other modern method.

### Indicators

We examined the readiness scores and coverage cascade of FP services. We calculated service readiness scores from the SPA using FP tracer indicators identified according to the service availability and readiness assessment (SARA) guidelines, a tool for assessing and monitoring the service availability and readiness of the health sector [24]. Based on the SARA, we classified the tracer indicators into four domains: availability, staff and guidelines, equipment, and medicines and commodities (**Table 2**). Availability referred to whether a facility provides or refers for all modern FP methods. Staff and guidelines related to whether guidelines were observed and staff received FP-related training. Equipment included blood pressure cuffs, and commodities included combined oral contraceptives, progestin-only pills, and injectable contraceptives (category 1), intrauterine devices and implants (category 2), male condoms (category 3), and emergency contraceptive pills (category 4). For Haiti, Nepal, and Tanzania, we removed the tracer indicator progestin-only contraceptives from the medicine and commodities domain because fewer than 30% of facilities in each country had them in stock, and we deduced that these commodities were not a key part of the national programme. Each domain was a binary or weighted average (0-1), with the total service readiness score a weighted average (**Table 3**).

**Table 2:** Domains and their tracer indicators of family planning service readiness

Domain/tracer indicators	Definition	Calculation method
Availability		
Modern contraceptive mix	Facility provides/refers for all modern methods: COC, POP, one-month injectable contraceptive, three-month injectable contraceptive, implant, male condom, IUD, emergency contraceptive pill, sterilisation, and standard days method	Each modern method that was provided or referred by a facility was categorised as 1, otherwise as 0. This domain was calculated by averaging the total value of the sum of modern methods categorised as 1.
Staff and guidelines		
*Guidelines on FP*	National or other FP guidelines observed in facility	A facility that observed either national or other FP guidelines was coded as 1, otherwise as 0. If the facility had more than 0 staff who received FP training and no missing staff, staff trained in FP were coded as 1, otherwise coded as 0. This domain was categorised as 1 when both guidelines on FP and staff trained in FP = 1. If either guidelines on FP or staff trained in FP = 0, this domain was categorised as 0.
*FP checklists or job aids*	Not available	
*Staff trained in FP*	Staff received in-service or updated training on FP-related topics within last two years	
Equipment		
*Blood pressure apparatus*	Blood pressure apparatus observed and functioning anywhere in facility	This domain was categorized as 1 if a blood pressure cuff was observed and functioning anywhere in the facility. Otherwise, it was categorised as 0.
Medicine and commodities		
*Category 1 (COC, POP, three-month injectable contraceptive)*	At least one valid dose observed available in facility on the day of assessment	Each item observed to have at least 1 valid dose available was categorised as 1. This domain was categorised as 1 only if all four commodities were categorised as 1. If the facility had none or some of the commodities, this domain was categorised as 0.
*Category 2 (IUD, implant)*	At least one valid dose observed available in facility on the day of assessment	
*Category 3 (male condom)*	At least one valid dose observed available in facility on the day of assessment	
*Category 4 (emergency contraceptive pill)*	At least one valid dose observed available in facility on the day of assessment	

COC – combined oral contraceptive, FP – family planning, IUD – intrauterine device, POP – progestin-only pill

**Table 3:** Service readiness component scores

		Bangladesh (mean, 95% CI)	Haiti (mean, 95% CI)	Malawi (mean, 95% CI)	Nepal 2015 (mean, 95% CI)	Nepal 2021 (mean, 95% CI)	Tanzania (mean, 95% CI)
**Domain**	**Total score**	0.58 (0.56–0.60)	0.65 (0.64–0.67)	0.66 (0.64–0.67)	0.59 (0.58–0.61)	0.60 (0.59–0.62)	0.65 (0.63–0.67)
Availability	Availability of modern methods	0.65 (0.63–0.67)	0.60 (0.58–0.61)	0.59 (0.58–0.60)	0.57 (0.55–0.59)	0.60 (0.59–0.62)	0.52 (0.50–0.54)
Equipment	Funx BP cuff anywhere in clinic	0.88 (0.85–0.91)	0.99 (0.98–1.00)	0.86 (0.84–0.89)	0.97 (0.95–0.99)	1.00 (0.99–1.00)	0.89 (0.86–0.92)
Staff and Guidelines	FP staff OR guidelines available	0.61 (0.56–0.65)	0.74 (0.71–0.77)	0.78 (0.75–0.80)	0.58 (0.53–0.62)	0.35 (0.32–0.39)	0.78 (0.74–0.82)
	FP guidelines	0.48 (0.44–0.53)	0.58 (0.54–0.61)	0.57 (0.54–0.60)	0.40 (0.36–0.45)	0.21 (0.17–0.24)	0.68 (0.64–0.73)
	FP-trained staff	0.31 (0.27–0.35)	0.46 (0.43–0.50)	0.52 (0.48–0.56)	0.32 (0.28–0.36)	0.21 (0.18–0.24)	0.39 (0.35–0.43)
Medicine and Commodities	All three/four categories stocked	0.19 (0.16–0.21)	0.29 (0.25–0.32)	0.40 (0.37–0.44)	0.26 (0.22–0.29)	0.46 (0.42–0.49)	0.42 (0.38–0.47)
	Category 1 (COC/POP/three-month injectable)	0.91 (0.88–0.93)	0.91 (0.89–0.93)	0.95 (0.94–0.97)	0.99 (0.98–0.99)	0.98 (0.97–0.99)	0.97 (0.95–0.98)
	Category 2 (IUD/implant)	0.24 (0.21–0.26)	0.30 (0.27–0.33)	0.67 (0.64–0.71)	0.26 (0.23–0.30)	0.47 (0.43–0.50)	0.55 (0.50–0.59)
	Category 3 (male condom)	0.85 (0.82–0.88)	0.85 (0.82–0.88)	0.77 (0.75–0.80)	0.98 (0.98–0.99)	0.97 (0.96–0.98)	0.77 (0.73–0.81)
	Category 4 (emergency)	0.42 (0.38–0.46)	N/A	0.54 (0.50–0.57)	N/A	N/A	N/A

BP – blood pressure, COC – combined oral contraceptive, FP – family planning, IUD – intrauterine device, POP – progestin-only pill

*N/A means the readiness score was too low to apply.

We applied the coverage cascade framework proposed by Amouzou and colleagues [11] in this analysis to quantify the use of FP services at different conditional stages. We focussed on service contact (women aged 15–49 who are currently using a modern method or women who met with a health worker or field worker for other care in the previous 12 months and mentioned FP), intervention coverage (demand for FP satisfied with modern methods), intervention coverage at a health facility (modern method was sourced at a health facility), and readiness-adjusted coverage (Table S1 in the **Online Supplementary Document**). Service contact, intervention coverage, and intervention coverage at a health facility were directly calculated from the DHS data. Readiness-adjusted coverage was estimated by linking data from the DHS and SPA using the ecological linking methodology, which uses facility type (*e.g.* hospital or clinic), managing authority (public or private), and administrative area (region or urban/rural locality) as the unit to link data from the DHS and SPA [25,26].

### Independent variables and data analysis

We categorised managing authority into public/government, private for-profit, and private not-for-profit (Table S3 in the **Online Supplementary Document**). We classified public facility types as hospital, health centre, health post/clinic, and community health workers. Private for-profit facilities included private hospitals, private health centres, private health posts/clinics, and private community health workers. We categorised any non-governmental organisation or mission/faith-based facility under a private not-for-profit managing authority. Sources such as pharmacies, shops, and churches were not considered formal sources, and quality was set to zero. Community health workers were not included in these SPAs, and we did not account for them in our analysis.

We categorised the geographical areas of Bangladesh, Haiti, and Tanzania into urban and rural because the number of facilities per linking unit was insufficient to generate a readiness score. We categorised Malawi into northern, central, and southern regions, and Nepal by ecological areas into mountain, hill, and *terai* (*i.e.* mountain base marshland). We performed a comparative analysis to determine disparities in FP service readiness and coverage by comparing the service readiness components and coverage cascade across the five countries. We then disaggregated the coverage cascade by geographical area (*i.e.* urban and rural), women’s age, and wealth quintile, and further analysed the absolute and relative gaps between intervention and readiness-adjusted coverage for an equity analysis. We calculated 95% confidence intervals (CIs) for the crude coverage and readiness scores. We used the sampling design and weights from the DHS to generate the standard error and 95% CIs for the readiness-adjusted indicators. The data were analysed with STATA, version 18.0 (StataCorp, College Station, Texas, USA).

## RESULTS

The most common contraceptive method varied by country (**Table 1**). Injectable contraceptives were the most prevalent in Haiti (50.0%), Malawi (49.0%), and Tanzania (30.6%). Oral contraceptives were most used in Bangladesh (41.0%), while sterilisation was the predominant method in Nepal, although its use decreased from 38.8% in 2015 to 30.2% in 2021 (**Table 1**). The source for modern contraceptives differed across countries, but the public sector was the main source (Table S4 in the **Online Supplementary Document**).

The national FP service readiness score ranged from 0.58 (95% CI = 0.56–0.60) in Bangladesh to 0.66 (95% CI = 0.64–0.67) in Malawi (**Table 3**). Bangladesh had the greatest availability of modern methods (0.65; 95% CI = 0.63–0.67), whereas availability in the other four countries ranged from 0.52 (95% CI = 0.50–0.54) in Tanzania to 0.60 in Haiti (95% CI = 0.58–0.61) and Nepal in 2021 (95% CI = 0.59–0.62). All countries had high availability of blood pressure cuffs in facilities (score of ≥0.86). Malawi (0.78; 95% CI = 0.75–0.80) and Tanzania (0.78; 95% CI = 0.74–0.82) had the highest score for FP staff or guidelines, whereas Nepal had the lowest in 2015 (0.58; 95% CI = 0.53–0.62) and 2021 (0.35; 95% CI = 0.32–0.39). Nepal had the highest stock of all four medical commodity categories in 2021 (0.46; 95% CI = 0.42–0.49), whereas Bangladesh had the lowest (0.19; 95% CI = 0.16–0.21). In Nepal, the overall service readiness score remained relatively consistent between surveys in 2015 (0.59; 95% CI = 0.58–0.61) and in 2021 (0.60; 95% CI = 0.59–0.62), but availability of contraceptives increased dramatically due to increased availability of intrauterine devices/implants from 0.26 (95% CI = 0.23–0.30) in 2015 to 0.47 (95% CI = 0.43–0.50) in 2021.

Among the five countries, public facilities had the highest levels of readiness (Table S5 in the **Online Supplementary Document**). However, the readiness levels of specific facility types varied by country and geographical area. In Haiti, Malawi, Nepal, and Tanzania, public hospitals had the highest readiness levels (0.79–0.91), regardless of the geographical area. In Bangladesh, the readiness levels were highest in public *upazila* health complexes, with scores of 0.90 and 0.94.

**Figure 1:**
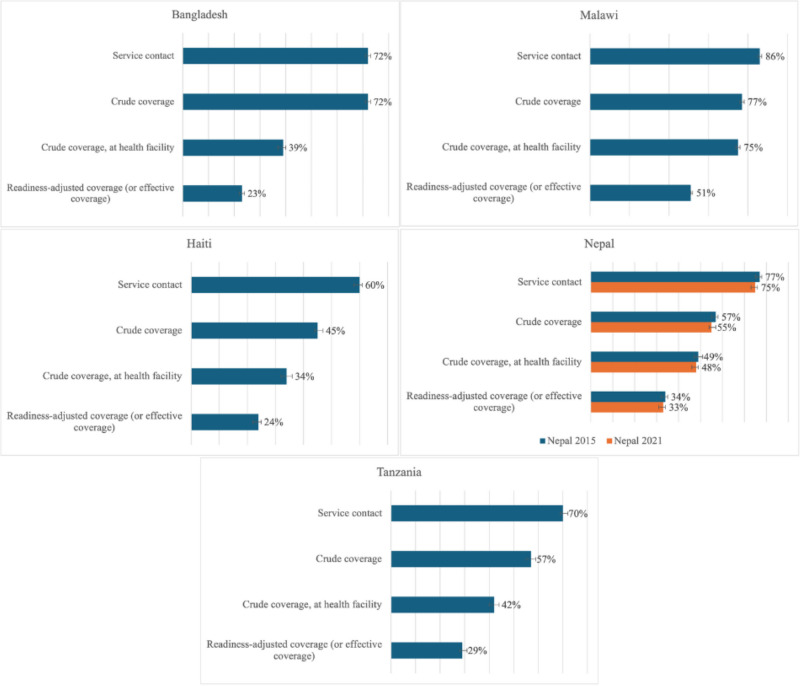
Family planning national coverage cascade for Bangladesh, Haiti, Malawi, Nepal, and Tanzania, with 95% confidence intervals. **Panel A.** Bangladesh. **Panel B.** Malawi. **Panel C.** Haiti. **Panel D.** Nepal. **Panel E.** Tanzania.

The percentage of women in need of FP services who had contact with FP services ranged from 60% (95% CI = 58–61) in Haiti to 86% (95% CI = 85–87) in Malawi (**Figure 1****,** Panels A–E). The proportion of women who met their FP needs with modern contraceptives from any source varied from 45% (95% CI = 44–47) in Haiti to 77% (95% CI = 76–78) in Malawi. The percentage of women who obtained modern contraceptives from formal health sectors ranged from 34% (95% CI = 32–36) in Haiti to 75% (95% CI = 74–76) in Malawi. In all countries, the readiness-adjusted coverage was much lower, with a median 24.5 percentage points (pp) difference between intervention and readiness-adjusted coverage in the five countries.

**Figure 2:**
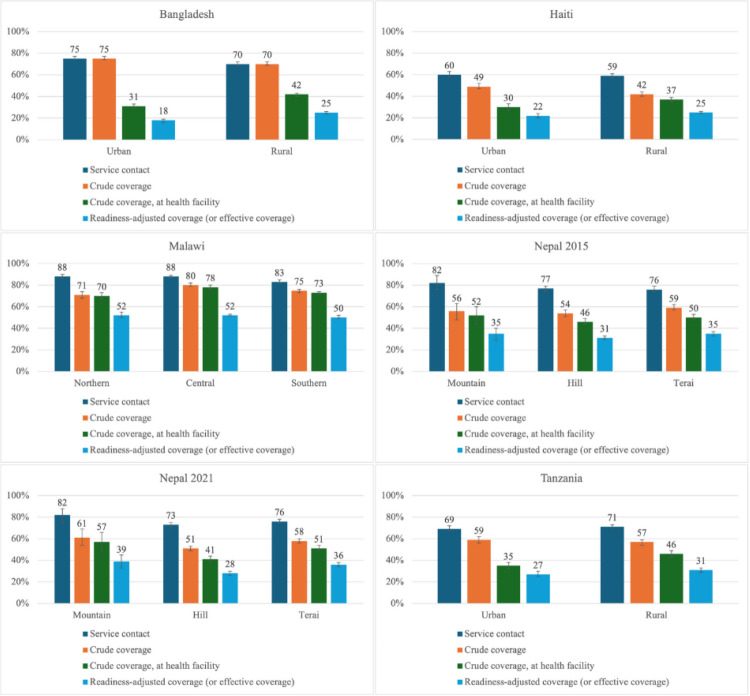
Family planning coverage cascade by geographic area for Bangladesh, Haiti, Malawi, Nepal, and Tanzania, with 95% confidence intervals. **Panel A.** Bangladesh. **Panel B.** Haiti. **Panel C.** Malawi. **Panel D.** Nepal 2015. **Panel E.** Nepal 2021. **Panel F.** Tanzania.

We observed little variation across urban and rural localities in Bangladesh, Haiti, and Tanzania and across geographic regions within Malawi and Nepal (**Figure 2****,** Panels A–F). In Bangladesh, Haiti, and Tanzania, intervention coverage was slightly higher in urban than in rural areas, but readiness-adjusted coverage was lower in urban compared to rural areas. Many women in urban areas obtain their contraceptives outside the formal health sector. Bangladesh had the largest intervention-readiness-adjusted gaps in both urban (57 pp; 76% relative reduction (rr)) and rural areas (45 pp; 64% rr). In Malawi, the absolute and relative gaps were similar across regions (19–28 pp; 27–35% rr). Nepal showed more similar absolute and relative gaps, with little change between 2015 (21–24 pp; 38–43% rr) and 2021 (22–23 pp; 36–45% rr).

**Figure 3:**
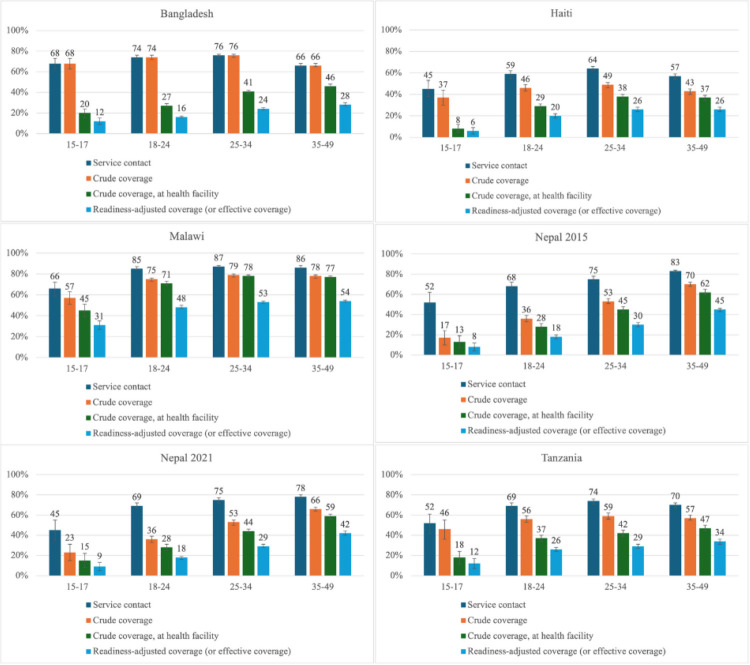
Family planning coverage cascade by women’s and girls’ age for Bangladesh, Haiti, Malawi, Nepal, and Tanzania, with 95% confidence intervals. **Panel A.** Bangladesh. **Panel B.** Haiti. **Panel C.** Malawi. **Panel D.** Nepal 2015. **Panel E.** Nepal 2021. **Panel F.** Tanzania.

Coverage at each stage of the cascade was lower for adolescents (15–17 years) than for the other three adult age groups (18–24, 25–34, and 35–49 years) (**Figure 3**, Panels A–F). The exception was Bangladesh, where service contact and intervention coverage for adolescents (68%; 95% CI = 63–73) was similar to that of the 35–49 age group (66%; 95% CI = 65–68). Crude coverage for adolescents ranged from 17% (95% CI = 10–24) in Nepal in 2015 to 68% (95% CI = 63–73) in Bangladesh, and readiness-adjusted coverage ranged from 6% (95% CI = 3–9) in Haiti to 31% (95% CI = 27–35) in Malawi. Adolescents had the largest relative gaps, with Haiti showing the largest reduction between crude and readiness-adjusted coverage (84% rr), followed by Bangladesh (82% rr), Tanzania (74% rr), Nepal (2015/2021 = 61%/53% rr), and Malawi (46% rr). Most adolescent girls obtained their contraceptives from outside of health facilities.

We also found that the wealthiest women had higher crude coverage in Haiti (49%; 95% CI = 45–52), Malawi (78%, 95% CI = 76–80), and Tanzania (59%; 95% CI = 55–62) compared to the poorest women in Haiti (37%; 95% CI = 34–41), Malawi (74%; 95% CI = 72–77), and Tanzania (43%; 95% CI = 39–47) (Figure S1 in the **Online Supplementary Document**). However, in Bangladesh, the wealthiest women (69%; 95% CI = 67–71) had lower intervention coverage than the poorest women (77%; 95% CI = 75–79). A similar pattern was observed in Nepal. When we adjusted for facility service readiness, the wealthiest women in Malawi and Tanzania had similar readiness-adjusted coverage as the poorest women. In contrast, in the other three countries, the wealthiest women had lower readiness-adjusted coverage than the poorest women, again because wealthier women sourced contraceptives from outside of health facilities. Among the five countries, Bangladesh had the largest intervention-readiness-adjusted coverage gaps for the wealthiest women (56 pp; 81% rr). In Nepal, the gap was half of the intervention coverage and was maintained between 2015 and 2021.

## DISCUSSION

We analysed FP service readiness and readiness-adjusted coverage and equity at facilities in Bangladesh, Haiti, Malawi, Nepal, and Tanzania. Other studies have described FP service readiness using these data sets, but ours is the first to focus on the coverage cascade by linking health facility and household surveys to describe the gaps and equity across a variety of settings [27,28]. We found that among all facility types, public hospitals had the highest readiness scores across the five countries, consistent with findings from previous studies [27–29]. Public hospitals might have greater readiness because they receive substantial investment from government sources and are required to comply with government policies regarding FP readiness. Additionally, hospitals tend to be located in urban areas and are better equipped and staffed than health centres [30].

Bangladesh had the largest coverage-readiness gap. Despite relatively high intervention coverage in Bangladesh, the large gap was driven by two factors. First, a substantial number of women obtained their contraceptives outside of health facilities. Second, facilities offering FP services were not always ready. In Malawi, on the other hand, most women obtained contraceptives from health facilities. Therefore, the gap between intervention coverage and readiness-adjusted coverage was due to low facility readiness for FP service. Studies have shown that the choices women make for obtaining FP services are often influenced by geographic distance and cost, and that women prefer facilities that are closest to their residence and offer free contraceptives [31–33].

We found that women living in urban areas of Bangladesh, Haiti, and Tanzania had higher intervention coverage than women living in rural areas. This finding was consistent with results from a previous study [34]. Surprisingly, readiness-adjusted coverage was lower for women living in urban areas compared to those living in rural areas; however, readiness scores of facilities were similar in urban and rural areas. Therefore, these differences may result from urban women relying on informal facilities for contraceptives (Table S6 in the **Online Supplementary Document**). For example, in Haiti, 37% of women in urban areas obtained their contraceptives from informal health facilities, compared to only 12% of women in rural areas.

Our study also revealed that adolescents were much more likely than adults to obtain modern contraceptives outside of health facilities (Table S6 in the **Online Supplementary Document**). Previous studies have shown that lack of privacy and confidentiality, as well as discriminatory and judgmental attitudes of healthcare providers, were major barriers to adolescents accessing contraception from health facilities [35–38].

### Limitations

One of the limitations of our study was the temporal difference between the DHS and the SPA data. For example, the Malawi SPA data were collected from July 2013 to February 2014, whereas the Malawi DHS data were collected between October 2015 and February 2016. The service readiness of facilities may have changed between those periods. Another limitation was the lack of consensus on definitions and measurements of facility readiness. The SARA tracer items have been used in other studies, but no linkage between service readiness and FP outcomes has been demonstrated. We employed an ecological linking method to link household and facility survey data, which connected a woman to an average readiness score but did not represent the readiness of the visited facility. Nevertheless, some studies have reported that ecological linking can produce outputs similar to those of direct linking [39]. Finally, these facility survey assessments did not include community-based health workers or private pharmacies. These health service delivery points may offer high levels of service readiness, but since we lacked data, we assumed a readiness of zero.

## CONCLUSIONS

Health facilities in Bangladesh, Haiti, Malawi, Nepal, and Tanzania showed moderate and variable facility readiness levels for providing FP services. This finding suggests that many women accessing health facilities for FP services might not receive the quality of care expected. Improving readiness-adjusted coverage requires not only enhancing the readiness of health facilities, but also addressing barriers that hinder women, especially adolescents, from accessing modern contraceptives. Efforts should focus on promoting health equity to ensure that all women, regardless of socioeconomic status, have equal access to high-quality FP services.

## Additional Material


Online Supplementary Document
