# Supplementary material for: Inequities in effective coverage of family planning services in low- and middle-income countries: linking household and facility surveys
Source: J Glob Health. 2025 Sep 12;15:04211. doi: 10.7189/jogh.15.04211 (PMC12427599; doi:10.7189/jogh.15.04211)
Supplement: Online Supplementary Document [file jogh-15-04211-s001.pdf]

Table S1. Coverage cascade and definitions

**Coverage cascade**	**Definition**
Services contact	A woman aged 15 to 49 who is currently using a modern method *or* woman who met with a health worker at a facility for other care or with a field worker in the previous 12 months and mentioned FP
Intervention coverage	A woman aged 15 to 49 in need of contraceptives who is currently using a modern contraceptive from any source
Intervention coverage at health facility	A woman aged 15 to 49 in need of contraceptives who is currently using a modern contraceptive sourced at a health facility (excludes private pharmacies, shops, etc.)
Readiness-adjusted coverage	A woman who is currently using modern contraceptives obtained at a health facility equipped to provide contraceptive services

Table S2. Family planning coverage cascade by quantiles of household asset score (data also presented in Figure S4)

	Bangladesh (%, 95% CI)		Haiti (%, 95% CI)		Malawi (%, 95% CI)		Nepal 2015 (%, 95% CI)		Nepal 2021 (%, 95% CI)		Tanzania (%, 95% CI)	
	Richest	Poorest	Richest	Poorest	Richest	Poorest	Richest	Poorest	Richest	Poorest	Richest	Poorest
Service contact	69 (67-71)	77 (75-79)	60 (56-64)	55 (51-59)	85 (83-86)	84 (83-86)	72 (68-75)	81 (78-84)	64 (60-67)	82 (80-85)	68 (65-71)	63 (59-68)
Crude coverage	69 (67-71)	77 (75-79)	49 (45-52)	37 (34-41)	78 (76-80)	74 (72-77)	53 (50-57)	55 (51-60)	50 (46-53)	57 (54-60)	59 (55-62)	43 (39-47)
Crude coverage, at health facility	23 (21-25)	51 (48-53)	24 (21-26)	36 (32-39)	74 (72-76)	73 (71-75)	39 (35-43)	51 (47-56)	34 (31-37)	53 (50-56)	32 (29-36)	38 (33-42)
Readiness-adjusted coverage (or effective coverage)	13 (12-14)	32 (30-33)	17 (15-19)	24 (22-26)	52 (50-53)	50 (48-51)	27 (25-30)	33 (30-36)	24 (21-26)	35 (33-37)	25 (22-28)	25 (22-28)
Crude-readiness-adjusted coverage absolute gap (percentage point (pp))	56	45	32	13	26	24	26	22	26	22	34	18
Crude-readiness-adjusted coverage relative gap (%)	-81	-58	-65	-35	-33	-32	-49	-40	-52	-39	-58	-42

Figure S1. Family planning coverage cascade by quantiles of household asset score for Bangladesh, Haiti, Malawi, Nepal, and Tanzania, with 95% confidence intervals. Panel A. Bangladesh. Panel B. Haiti. Panel C. Malawi. Panel D. Nepal 2015. Panel E. Nepal 2021. Panel F. Tanzania.

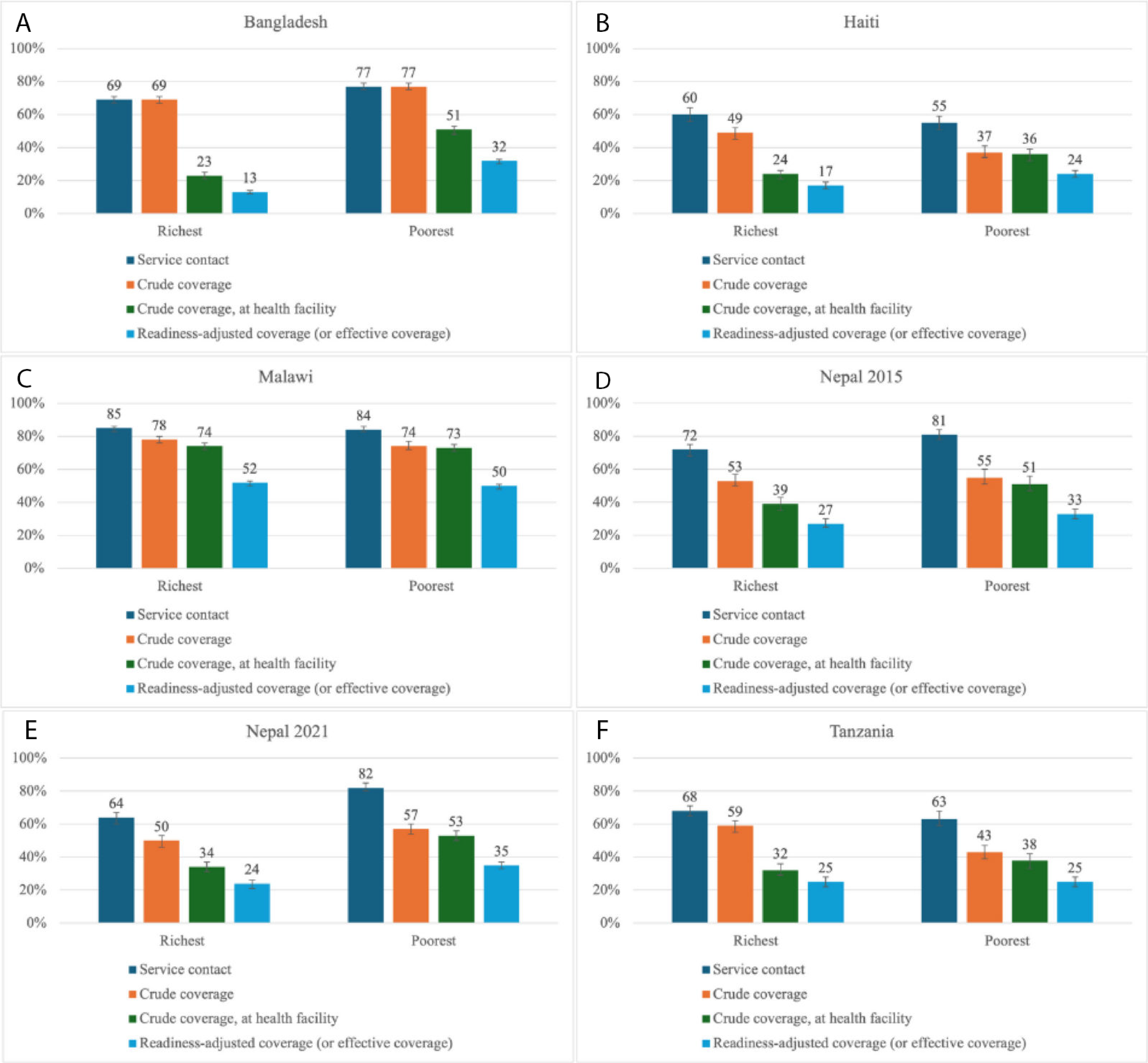

Table S3. Provider Crosswalk

			SPA 2017	DHS 2017-18
Bangladesh	Public	Government/public hopistal	district hospital	medical college hospital
				specialized govt hospital
				district hospital
		Government/public UHC	upazila health complex (UHC)	upazila health complex
		Government/public MCWC	Mother and Child Welfare Center (MCWC)	Mother and Child Welfare Center (MCWC)
		Government/public UnHFWC	union health and family welfare center (UnHFWC)	union health and family welfare center
			union health and family welfare center (UnHFWC - Upgraded)	
			union subcenter (UnSC) / rural dispensary	
		Government/public health clinic/post	community clinic	community clinic
				satellite clinic/epi outreach
				other public sector
				government field worker (fwa)
	Private	Private for profit	private hospital	private medical college hospital
				private hospital
				private clinic
				qualified doctor's chamber
				non-qualified doctor's chamber
		Private for non-profit (local govt/NGO)	ngo clinic (other than smiling sun clinics)	ngo static clinic
			ngo hospital	ngo satellite clinic
			smiling sun clinic of ngo health service delivery project (NHSDP)	ngo depo holder
				ngo field worker
				other ngo sector
	Not a health faciliy	Not a health faciliy		pharmacy/drug store
				shop
				friend/relative

				other
				don't know

			SPA 2017-18	DHS 2016-17
**Haiti**	Public	Gorvenment/public hospital	university hospital	government hospital
			department hospital	mix hospital/clinic
			community reference hospital	
			other hospitals	
		Gorvenment/public health center	health center with lit	government health center
			health center without lit	mix health center
		Gorvenment/public health clinic/post	dispensary/community health center	mix family planning clinic
	Private	Private for profit	university hospital	private hospital, clinic
			community reference hospital	private health center
			other hospitals	family planning clinic
			health center with lit	private doctor
			health center without lit	
			dispensary/community health center	
		Private for non-profit	department hospital	mobile clinic (ngo)
			other hospitals	health worker (ngo)
			health center with lit	
			health center without lit	
			dispensary/community health center	
	Not a health facility	Not a health facility		pharmacy
				shop
				slot-machine
				firend/relative
				other

			SPA 2013-14	DHS 2015-16
**Malawi**	Public	Government/public hopistal	central hospital	government hospital
			district hospital	
			government/public rural/community hospital	
			government/public other hospital	
		Government/public health center	government/public health center	government health center
		Government/public health clinic/post	government/public maternity	government health post/outreach
			government/public dispensary	mobile clinic
			government/public clinic	hsa
			government/public health post	cbda/door to door
	Private	Private for profit	private other hospital	private hospital/clinic
			private health centre	private mobile clinic
			private clinic	prvate cbda/door to door
			private maternity	private doctor
			private dispensary	
			company health centre	
			company dispensary	
			company clinic	
		Private for non-profit	cham rural/community hospital	cham/mission hospital
			cham other hospital	cham/mission health center
			cham health centre	cham/mission mobile clinic
			cham maternity	cham/clinic door to door
			cham dispensary	blm
			cham clinic	macro
			mission/faith-based other hospital	youth drop in center
			mission/faith-based clinic	
			ngo health centre	
			ngo clinic	

	Not a health faciliy	Not a health faciliy		other private medical<br>pharmacy<br>shop<br>church<br>friend/relative<br>other<br>don't know

**Nepal**			**SPA 2015**	**DHS 2016**
	Public	Gorvenment/public hospital	central government hospital<br>regional government hospital<br>sub-reginal government hospital<br>zonal government hospital<br>district government hospital<br>other public hopital<br>central level government hospital<br>district level government hospital	government hospital/clinic
		Gorvenment/public health center	primary health care center<br>urban health centre	primary health care center
		Gorvenment/public health clinic/post	health post<br>sub-health post	health post/sub health post<br>primary health care outreach clinic<br>mobile camp<br>female community health volunteer<br>other public facilities<br>institutionalized fp clinics
	Private	Private for profit	other hospital	private hospital/nuring home<br>private clinic<br>sangini outlet
		Private for non-profit	other hospital<br>htc	family planning association of nepal<br>marie stopes

				other ngo facilities
	Not a health facility	Not a health facility		other private medical facilities
				pharmacy
				shop
				firend/relative
				other
				don't know
			**SPA 2021**	**DHS 2022**
	Public	Gorvenment/public hospital	federal level hospital	government hospital
			provincial level hospital	phc/primary hospital
			local level hospital	
		Gorvenment/public health center	primary health care center (phcc)	basic health care center
			community health unit (chu)	community health unit
			urban health centre (uhc)	urban health center
		Gorvenment/public health clinic/post	health post (hp)	health post
				mobile camp
				female community health volunteer (fchv)
				other public sector
				institutionalized fp clinics
	Private	Private for profit	other hospital	private hospital
				private clinic
				sangini outlet
		Private for non-profit, mission/faith-based	other hospital	family planning association of nepal (fpan)
			htc	marie stopes
				other ngo medical sector
	Not a health facility	Not a health facility		other private medical sector
				pharmacy

				shop
				firend/relative
				other
				don't know

			SPA 2014-15	DHS 2015-16
**Tanzania**	Public/Parastatal	Government/public/parastatal hopistal	national referral hospital	national/zonal referral/spec. hospital (government/parastatal)
			regional hospital	regional referral hospital (government/parastatal)
			district hospital	regional hospital (government/parastatal)
			district-designated hospital	district hospital (government/parastatal)
			other hospital (private)	
		Government/public/parastatal health center	health centre	health center (government/parastatal)
		Government/public/parastatal health clinic/dispensary	clinic	dispensary (government/parastatal)
			dispensary	clinic (government/parastatal)
				chw (government/parastatal)
	Private	Private for profit	other hospital (private)	specialized hospital (private)
			health centre	hospital (private)
			clinic	health centre (priavte)
			dispensary	dispensary (priavte)
				clinic (private)
		Mission/faith-based	national referral hospital	referal/sepc. hospital (religious/voluntary)
			regional hospital	district hospital (religious/voluntary)
			district hospital	hospital (religious/voluntary)
			district-designated hospital	health centre (religious/voluntary)
			other hospital (private)	dispensary (religious/voluntary)
			health centre	clinic (religious/voluntary)
			clinic	ngo
			dispensary	

	Not a health faciliy	Not a health faciliy	pharmacy
			acredited drug dispensing outlet (addo)
			shop/kiosk
			bar
			guest house/hotel
			friend/relative/neighor
			vct centre
			other

Table S4. Formal Source for Modern Method

Country	Geographic area, Facility type	Proportion	Sample Size
Bangladesh	urban, public hosp	1%	111
	urban, public UHC	4%	251
	urban, public MCWC	1%	107
	urban, public UnHFWC	1%	76
	urban, public clinic/post	9%	587
	urban, private for profit	3%	182
	urban, private for non-profit	4%	355
	rural, public hosp	2%	120
	rural, public UHC	12%	559
	rural, public MCWC	2%	108
	rural, public UnHFWC	7%	345
	rural, public clinic/post	43%	2010
	rural, private for profit	6%	262
	rural, private for non-profit	4%	203
Haiti	Urban, public hosp	12%	254
	Urban, public HC	16%	315
	Urban, public clinic/post	1%	20
	Urban, private for profit	6%	111
	Urban, private for non-profit	5%	122
	Rural, public hosp	14%	353
	Rural, public HC	26%	709
	Rural, public clinic/post	1%	15
	Rural, private for profit	5%	133
	Rural, private for non-profit	15%	417
Malawi	North, public hosp	2%	561
	North, public HC	6%	768
	North, public clinic/post	2%	250
	North, private for profit	0%	29
	North, private for non-profit	1%	440
	Central, public hosp	7%	822
	Central, public HC	24%	1,870

	Central, public clinic/post	6%	468
	Central, private for profit	3%	218
	Central, private for non-profit	5%	435
	South, public hosp	6%	999
	South, public HC	23%	2,480
	South, public clinic/post	5%	512
	South, private for profit	3%	238
	South, private for non-profit	6%	600
**Nepal 2016**	Mountain, public hosp	2%	80
	Mountain, public HC	0%	19
	Mountain, public post	4%	168
	Mountain, private for profit	0%	7
	Mountain, private for non-profit	0%	3
	Hill, public hosp	12%	483
	Hill, public HC	1%	53
	Hill, public post	19%	802
	Hill, private for profit	6%	188
	Hill, private for non-profit	3%	78
	Terai, public hosp	23%	794
	Terai, public HC	2%	84
	Terai, public post	16%	625
	Terai, private for profit	6%	204
	Terai, private for non-profit	4%	137
**Nepal 2022**	Mountain, public hosp	2%	139
	Mountain, public HC	1%	54
	Mountain, public post	4%	258
	Mountain, private for profit	0%	14
	Mountain, private for non-profit	0%	7
	Hill, public hosp	10%	465
	Hill, public HC	2%	103
	Hill, public post	17%	965
	Hill, private for profit	5%	170
	Hill, private for non-profit	1%	69

	Terai, public hosp	20%	713
	Terai, public HC	1%	36
	Terai, public post	24%	942
	Terai, private for profit	9%	315
	Terai, private for non-profit	4%	142
**Tanzania**	Mainland urban, public/parastatal hosp	9%	194
	Mainland urban, public/parastatal HC	8%	171
	Mainland urban, public/parastatal clinic/dispensary	8%	186
	Mainland urban, private for profit	4%	67
	Mainland urban, mission/faith-based	3%	72
	Mainland rural, public/parastatal hosp	6%	147
	Mainland rural, public/parastatal HC	11%	223
	Mainland rural, public/parastatal clinic/dispensary	42%	998
	Mainland rural, private for profit	2%	41
	Mainland rural, mission/faith-based	8%	162

Table S5. Readiness Score by Linking Unit

Country	Geographic area, Facility type	Readiness Score	Sample Size
**Bangladesh**	Urban, public hosp	0.74	51
	Urban, public UHC	0.94	72
	Urban, public MCWC	0.90	71
	Urban, public UnHFWC	0.87	5
	Urban, public clinic/post	0	0
	Urban, private for profit	0.49	57
	Urban, private for non-profit	0.85	68
	Rural, public hosp	0	0
	Rural, public UHC	0.90	62
	Rural, public MCWC	0.82	19
	Rural, public UnHFWC	0.76	600
	Rural, public clinic/post	0.49	302
	Rural, private for profit	0.58	4
	Rural, private for non-profit	0.75	42
**Haiti**	Urban, public hosp	0.86	45
	Urban, public HC	0.71	76
	Urban, public clinic/post	0.78	15
	Urban, private for profit	0.58	75
	Urban, private for non-profit	0.66	33
	Rural, public hosp	0.79	10
	Rural, public HC	0.68	119
	Rural, public clinic/post	0.64	211
	Rural, private for profit	0.58	111
	Rural, private for non-profit	0.59	61
**Malawi**	North, public hosp	0.85	13
	North, public HC	0.77	73
	North, public clinic/post	0.52	11
	North, private for profit	0.54	20
	North, private for non-profit	0.72	25
	Central, public hosp	0.81	16
	Central, public HC	0.69	131

	Central, public clinic/post	0.52	22
	Central, private for profit	0.58	84
	Central, private for non-profit	0.63	57
	South, public hosp	0.85	20
	South, public HC	0.72	139
	South, public clinic/post	0.45	32
	South, private for profit	0.57	108
	South, private for non-profit	0.71	59
**Nepal 2015**	Mountain, public hosp	0.91	17
	Mountain, public HC	0.78	23
	Mountain, public post	0.55	86
	Mountain, private for profit	0.48	5
	Mountain, private for non-profit	0.63	4
	Hill, public hosp	0.85	51
	Hill, public HC	0.70	117
	Hill, public post	0.59	192
	Hill, private for profit	0.61	45
	Hill, private for non-profit	0.72	22
	Terai, public hosp	0.82	33
	Terai, public HC	0.69	105
	Terai, public post	0.56	145
	Terai, private for profit	0.55	35
	Terai, private for non-profit	0.75	19
**Nepal 2021**	Mountain, public hosp	0.82	21
	Mountain, public HC	0.48	102
	Mountain, public post	0.68	62
	Mountain, private for profit	0.41	7
	Mountain, private for non-profit	0.36	1
	Hill, public hosp	0.84	71
	Hill, public HC	0.50	416
	Hill, public post	0.62	209
	Hill, private for profit	0.58	83
	Hill, private for non-profit	0.62	23

	Terai, public hosp	0.87	45
	Terai, public HC	0.53	235
	Terai, public post	0.62	109
	Terai, private for profit	0.56	73
	Terai, private for non-profit	0.70	21
**Tanzania**	Mainland urban, public/parastatal hosp	0.91	92
	Mainland urban, public/parastatal HC	0.85	56
	Mainland urban, public/parastatal clinic/dispensary	0.65	30
	Mainland urban, private for profit	0.80	54
	Mainland urban, mission/faith-based	0.65	35
	Mainland rural, public/parastatal hosp	0.79	19
	Mainland rural, public/parastatal HC	0.79	197
	Mainland rural, public/parastatal clinic/dispensary	0.61	298
	Mainland rural, private for profit	0.56	17
	Mainland rural, mission/faith-based	0.70	56

Table S6. Informal Source for Modern Method by Geographic Area, Women's age, and Household Wealth

Country		Sample size	Proportion
Bangladesh	Urban	2097	56%
	Rural	2505	41%
	15-17	188	71%
	18-24	1455	62%
	25-34	1930	47%
	35-49	1029	32%
	Poorest	718	36%
	Richest	1365	65%
Haiti	Urban	487	37%
	Rural	215	12%
	15-17	72	76%
	18-24	308	37%
	25-34	223	18%
	35-49	99	10%
	Poorest	37	6%
	Richest	267	48%
Malawi	Northern	38	2%
	Central	101	3%
	Southern	141	3%
	15-17	49	20%
	18-24	133	5%
	25-34	64	1%
	35-49	34	1%
	Poorest	28	2%
	Richest	136	5%

**Nepal 2015**	Mountain	21	7%
	Hill	237	13%
	Terai	322	15%
	15-17	8	29%
	18-24	102	20%
	25-34	241	15%
	35-49	229	10%
	Poorest	68	8%
	Richest	186	26%
**Nepal 2021**	Mountain	25	5%
	Hill	300	14%
	Terai	276	11%
	15-17	9	35%
	18-24	104	19%
	25-34	234	13%
	35-49	254	10%
	Poorest	107	8%
	Richest	176	29%
**Tanzania**	Mainland Urban	396	36%
	Mainland Rural	334	18%
	15-17	41	58%
	18-24	231	31%
	25-34	288	26%
	35-49	170	16%
	Poorest	42	11%
	Richest	311	42%
